# Analytical solution for vacuum preloading considering the nonlinear distribution of horizontal permeability within the smear zone

**DOI:** 10.1371/journal.pone.0139660

**Published:** 2015-10-08

**Authors:** Jie Peng, Xiang He, Hanming Ye

**Affiliations:** Research Institute of Geotechnical Engineering, Hohai University, Nanjing 210098, PR China; University of Aveiro, PORTUGAL

## Abstract

The vacuum preloading is an effective method which is widely used in ground treatment. In consolidation analysis, the soil around prefabricated vertical drain (PVD) is traditionally divided into smear zone and undisturbed zone, both with constant permeability. In reality, the permeability of soil changes continuously within the smear zone. In this study, the horizontal permeability coefficient of soil within the smear zone is described by an exponential function of radial distance. A solution for vacuum preloading consolidation considers the nonlinear distribution of horizontal permeability within the smear zone is presented and compared with previous analytical results as well as a numerical solution, the results show that the presented solution correlates well with the numerical solution, and is more precise than previous analytical solution.

## Introduction

In recent decades, the construction of infrastructure over soft soil has necessitated advances in soil improvement techniques. The drainage consolidation method is widely used in soft ground treatment. In drainage consolidation, prefabricated vertical drains (PVDs) are installed in the ground to accelerate the consolidation of the soil [1–5]. In practical engineering, PVDs have often been used together with vacuum preloading since Kjellman introduced the vacuum preloading method to improve soil strength[6–7]. The theory of radial consolidation was initially presented by Barron[1]. Subsequently, Yoshikuni et al.[8] proposed analytical solution included well resistance. Hansbo[2] and Onoue[9] extended these solutions to take the smear effect into account. In terms of vacuum preloading, Mohanmdelhassan et al.[10] proposed a rigorous solution for vertical consolidation and Indraratna et al.[11] presented a solution for radial consolidation. After those, several analytical solutions have been proposed in which the both vertical and radial drainage including well resistance and smear effect were considered to analyze the behavior of soil under vacuum preloading[12–16].

These analytical solutions mentioned above generally divide the soil around a drain well into two annular zones: smear and undisturbed. A reduced but constant horizontal permeability coefficient (*k*_*h*_) is adopted within the smear zone, and the vertical permeability coefficient (*k*_*v*_) remains constant. But the soil permeability coefficient in smear zone changes continuously in reality, and it is not appropriate to approximate the permeability coefficient distribution using two zones. Hossam et al.[17] proposed a solution that considers linear changes in the horizontal permeability when surcharge preloading was applied, and Walker et al.[18] considered the parabolic distribution of permeability within the smear zone under surcharge. To the authors’ knowledge, however, there is no analytical solution of vacuum preloading consolidation in which the nonlinear distribution of permeability in the smear zone is considered in the literature. This paper presents a solution for vacuum preloading consolidation with PVDs that is based on the equal strain hypothesis and the nonlinear decrease in the horizontal permeability coefficient (*k*_*h*_) toward the drain within the smear zone is considered. The proposed solution is assessed by comparing it with previous analytical and numerical solutions.

## Theoretical model

### Permeability of the PVD smear zone

**Fig 1:**
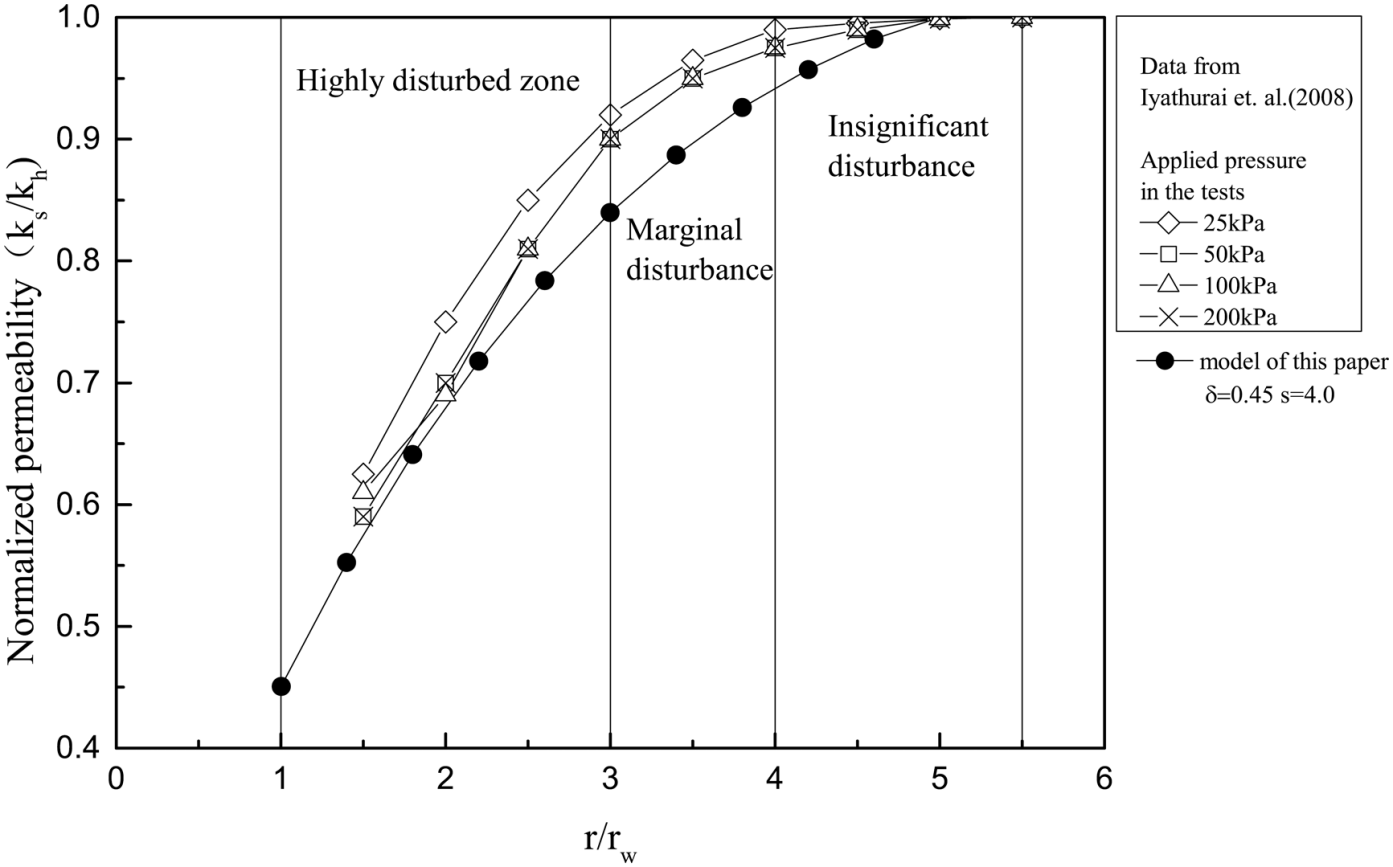
Smear zone permeability coefficient curve based on data from Iyathurai et al.[24] and the proposed model.

The smear zone is a disturbed area formed around a PVD during its installation by mandrel. The permeability in a smear zone is less than that in the surrounding undisturbed zone. The reduction in the permeability of the soil in the smear zone is called the smear effect. Many researchers [19–21] have noted that the disturbance in the smear zone increases toward the drain. The extent of smearing depends on the mandrel size and soil type[22,23] and is reported to vary from 1.2 to 7 based on theoretical and experimental studies[5]. The laboratory tests conducted by Indraratna et al. [13], Iyathurai et al.[24], and Sharma et al. [20] demonstrate that the horizontal permeability coefficient (*k*_*h*_) decreases substantially in a nonlinear manner toward the drain within the smear zone. Iyathurai et al.[24]documented the permeability coefficient in the smear zone, as shown in Fig 1. The existing literature suggests that the vertical permeability coefficient is almost unchanged within the smear zone[25].

To simulate the actual change in permeability within the smear zone, this study adopts an exponential function that is continuous, convex, and smooth in (*r*_*w*_,*r*_*e*_) and continuous at the point *r*_*s*_, as follows:

$$k_{h}\left(r\right)=f(r)\cdot k_{h}=a(\frac{r}{r_{w}}).e^{-\beta (\frac{r}{r_{s}})}\cdot k_{h}\begin{array}{cc} & \left(r_{w}\leq r\leq r_{s}\right) \end{array}$$ (1)

and

$$k_{h}\left(r\right)=k_{h}\begin{array}{cccc} & & & (r_{s}\leq r\leq r_{e} \end{array})$$

in which *r* is the radial position, *r*_*s*_ is the radius of the smear zone, *r*_*w*_ is the radius of the drain, *k*_*h*_ is the horizontal permeability coefficient of the undisturbed zone, and *a* and *β* are coefficients of this function that are determined by the following conditions:

$$r=r_{w},f(r_{w})=\delta =a\cdot e^{-\beta (\frac{1}{s})}$$ (2-a)

$$r=r_{s},f(r_{s})=1=a\cdot se^{-\beta }$$ (2-b)

where $s=\frac{r_{s}}{r_{w}}$ and $\delta =\frac{k_{h}\left(r_{w}\right)}{k_{h}}$, in which *k*_*h*_ *(r*_*w*_ *)* is the permeability coefficient at the drain-soil interface.

Thus,

$$\beta =\frac{s}{s-1}\text{ln}(s\delta )$$

$$a=\frac{1}{s}.e^{\beta }=\frac{1}{s}.{\left(s\delta \right)}^{\frac{s}{s-1}}$$

and the consolidation coefficients within the smear zone can be expressed as follows:

$$\left\{\begin{array}{l} C_{v}(r)=C_{v}=\frac{k_{v}}{\gamma_{w}m_{v}}\\ C_{h}(r)=f(r)C_{h}=\frac{f(r)k_{h}}{\gamma_{w}m_{h}} \end{array}\right\}$$ (3)

where $C_{v}=\frac{k_{v}}{\gamma_{w}m_{v}}$ and $C_{h}=\frac{k_{h}}{\gamma_{w}m_{v}}$ are the vertical and horizontal consolidation coefficients in the undisturbed zone, *k*_*v*_ is the vertical permeability coefficient in the undisturbed zone, *γ*_*w*_ is the specific weight of water, and *m*_*v*_ is the coefficient of the volume compressibility of the soil.

Based on the results of Iyathurai et al.[24], δ = 0.45 and s = 4.0 are substituted into Eq 1. The resulting curve for the permeability coefficient is given in Fig 1 and is close to that found by Iyathurai et al.[24]. This correlation demonstrates that the proposed exponential function model is suitable for simulating the nonlinear permeability change within the smear zone. Therefore, Eq 1 is adopted to simulate the nonlinear change of the permeability coefficient within the smear zone in this study's analytical model of vertical drain consolidation with vacuum preloading.

### Axisymmetric model

**Fig 2:**
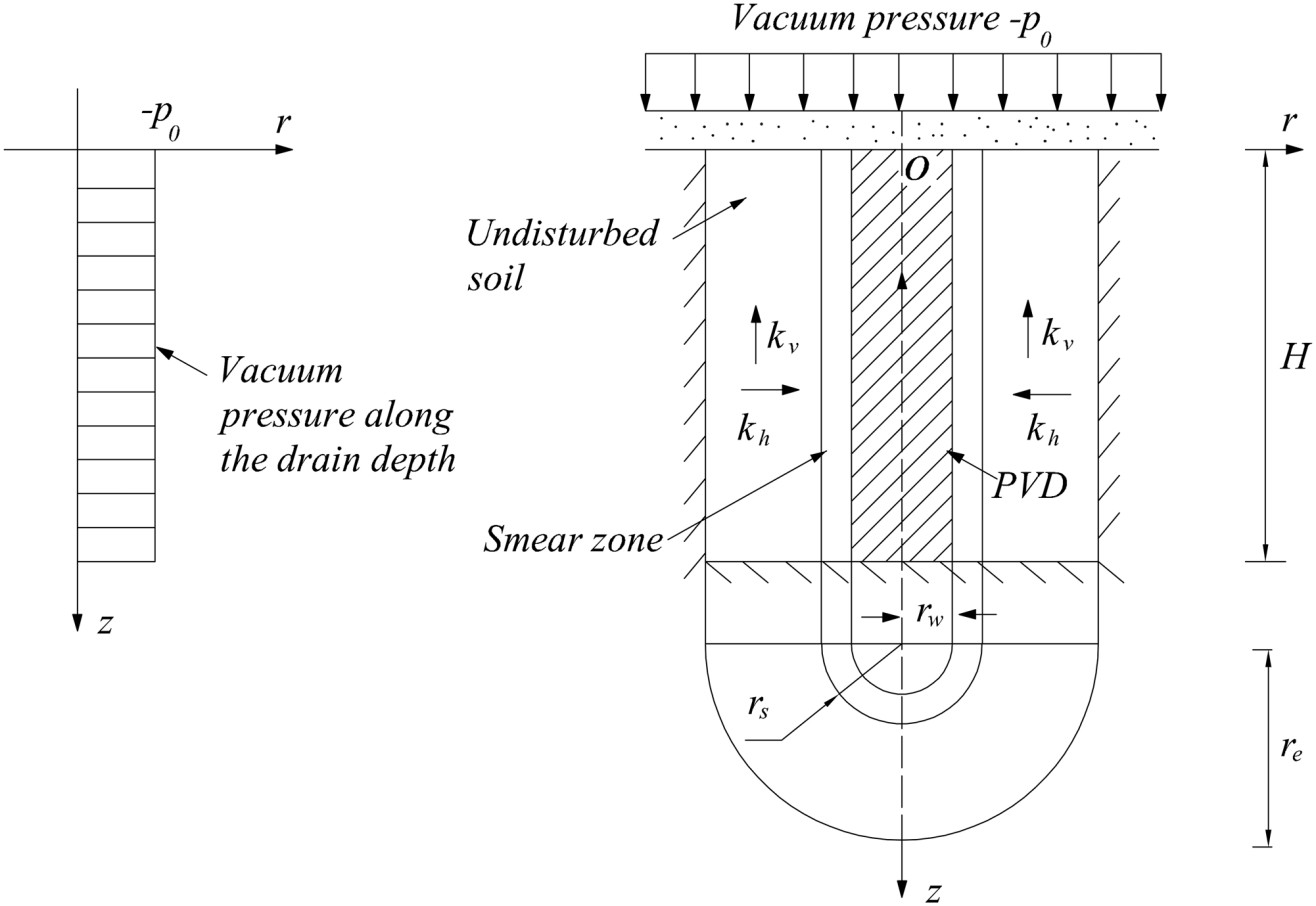
Schematic diagram of the vacuum preloading method with a PVD.

A schematic of the vacuum preloading method with a PVD is provided in Fig 2. The basic partial differential equation for vertical drain consolidation is as follows:

$$\frac{\partial u}{\partial t}-c_{h}\left(\frac{\partial^{2}u}{\partial r^{2}}+\frac{1}{r}\frac{\partial u}{\partial r}\right)-c_{v}^{}\frac{\partial^{2}u}{\partial z^{2}}=0$$ (4)

where *u*, *c*_*h*_, *c*_v,_ *t*, *r*, and *z* are the excess pore water pressure, the horizontal and vertical coefficients of consolidation, time, and the radial and vertical coordinates, respectively. The equal strain hypothesis is adopted, in which the vertical drain and the surrounding soils deform only vertically and have equal strains at any depth and radius. Thus, the basic equations for the smear zone and the undisturbed zone are as follows:

$$\begin{array}{l} L(u_{1})=\frac{\partial \bar{u}(z,t)}{\partial t}-c_{v}\frac{\partial^{2}\bar{u}(z,t)}{\partial z^{2}}-c_{h}\frac{1}{r}\left(\frac{\partial }{\partial r}[r\frac{\partial u_{1}(r,z,t)}{\partial r}]\right)=0\\ \begin{array}{cccc} & & & \begin{array}{ccc} & & \left(r_{s}\leq r\leq r_{e}\;0\leq z\leq H,t>0\right) \end{array} \end{array} \end{array}$$ (5-a)

$$\begin{array}{l} L(u_{2})=\frac{\partial \bar{u}(z,t)}{\partial t}-c_{v}(r)\frac{\partial^{2}\bar{u}(z,t)}{\partial z^{2}}-c_{h}(r)\frac{1}{r}\left(\frac{\partial }{\partial r}[r\frac{\partial u_{2}(r,z,t)}{\partial r}]\right)=0\\ \begin{array}{cccc} & & & \begin{array}{ccc} & & \left(r_{w}\leq r\leq r_{s}\;0\leq z\leq H,t>0\right) \end{array} \end{array} \end{array}$$ (5-b)

in which *u*_1_ and *u*_2_ are the excess pore water pressure of the smear and undisturbed zones, *c*_*v*_(*r*) and *c*_*h*_(*r*) are the consolidation coefficients in the smear zone, as given by Eq 3, and $\bar{u}$ is the average excess pore water pressure in the radial direction, calculated as follows:

$$\bar{u}(z,t)=\frac{1}{r_{e}^{2}-r_{w}^{2}}[\int_{r_{s}}^{r_{e}}u_{1}(r,z,t)dz+\int_{r_{w}}^{r_{e}}u_{2}(r,z,t)dz]$$ (6)

The effect of well resistance is neglected in this study because PVDs typically provide adequate discharge capacity in practical vacuum preloading engineering[26]. Therefore, the boundary conditions in the analysis are as follows:

$$u_{1}(r,0,t)=u_{2}(r,0,t)=u_{w}(r,0,t)=-p_{0}$$ (7)

$$\frac{\partial u_{1}}{\partial z}|{}_{Z=H}=\frac{\partial u_{2}}{\partial z}|{}_{Z=H}=\frac{\partial u_{w}}{\partial z}|{}_{Z=H}=0$$ (8)

$$\frac{\partial u}{\partial r}|{}_{r=r_{e}}=0$$ (9)

in which *u*_*w*_ is the excess pore water pressure in the drain and -*p*_*0*_ is the vacuum pressure.

The continuity conditions are as follows:

$$u_{2}(r_{w},z,t)=u_{w}(r_{w},z,t)=-p_{0}\begin{array}{cc} , & (t> \end{array}0)$$ (10)

$$u_{1}(r_{s},z,t)=u_{2}(r_{s},z,t)$$ (11-a)

$$\frac{\partial u_{1}}{\partial r}|{}_{r=r_{s}}=\frac{\partial u_{2}}{\partial r}|{}_{r=r_{s}}$$ (11-b)

When *t* = 0, the initial conditions are given by

$$u_{1}(r,z,0)=u_{2}(r,z,0)=u_{w}(r,z,0)=0$$ (12)

From the above equations, we obtain the average excess pore water pressure $\bar{u}(t)$ and the average consolidation degree *U*(*t*):

$$\text{Average excess pore water pressure }\bar{u}(t):\;\bar{u}(t)=\frac{1}{H}\int_{0}^{H}\bar{u}(z,t)dz$$ (13)

$$\text{Average consolidation degree }U(t):U(t)=\frac{\bar{u}(t)}{-p_{0}}$$ (14)

## Solution

Taking the integral of Eq 5-a from *r*_*e*_→*r* and using Eq 9, we have

$$\frac{\partial u_{1}(r,z,t)}{\partial r}=\frac{1}{2}\cdot \left(\frac{1}{c_{h}}\frac{\partial \bar{u}(z,t)}{\partial t}-\frac{c_{v}}{c_{h}}\frac{\partial^{2}\bar{u}(z,t)}{\partial z^{2}}\right)\left(r-\frac{r_{e}^{2}}{r}\right)$$ (15)

Taking the integral of Eq 15, again from *r*_*s*_→*r*, we have

$$\begin{array}{l} u_{1}(r,z,t)=\;u(r_{s},z,t)+\frac{1}{2}\left(\frac{1}{c_{h}}\frac{\partial \bar{u}(z,t)}{\partial t}-\frac{c_{v}}{c_{h}}\frac{\partial^{2}\bar{u}(z,t)}{\partial z^{2}}\right)\left\{\frac{1}{2}\left[r^{2}-r_{s}^{2}\right]-r_{e}^{2}\ln\left(\frac{r}{r_{s}}\right)\right\}\\ \begin{array}{ccccccccccccccc} & & & & & & & & & & & & & \left(r_{s}\leq r\leq r_{e};0\leq z\leq H;t>0\right) & \end{array} \end{array}$$ (16)

Taking the integral of Eq 5-b from *r*_*s*_→*r*, we have

$$r\frac{\partial u_{2}}{\partial r}={r_{s}\frac{\partial u_{2}}{\partial r}|}_{r=r_{s}}+\left(\frac{1}{c_{h}}\frac{\partial \bar{u}(z,t)}{\partial t}-\frac{c_{v}}{c_{h}}\frac{\partial^{2}\bar{u}(z,t)}{\partial z^{2}}\right)\cdot \int_{r_{s}}^{r}\frac{r}{f(r)}dr$$ (17)

in which

$$\int_{r_{s}}^{r}\frac{r}{f(r)}dr=\int_{r_{s}}^{r}\frac{r}{a(\frac{r}{r_{w}})\cdot e^{-\beta (\frac{r}{r_{s}})}}dr=\frac{s}{a\cdot \beta }r_{w}^{2}\left[e^{\beta (\frac{r}{r_{s}})}-e^{\beta }\right]$$ (18)

Combining Eqs (11-b) and (15) yields

$${r_{s}\frac{\partial u_{2}}{\partial r}|}_{r=r_{s}}={r_{s}\frac{\partial u_{1}}{\partial r}|}_{r=r_{s}}=\frac{1}{2}\cdot \left(\frac{1}{c_{h}}\frac{\partial \bar{u}(z,t)}{\partial t}-\frac{c_{v}}{c_{h}}\frac{\partial^{2}\bar{u}(z,t)}{\partial z^{2}}\right)r_{s}^{2}\left(1-\frac{n^{2}}{s^{2}}\right)$$ (19)

where $n=\frac{r_{e}}{r_{w}}$ and $s=\frac{r_{s}}{r_{w}}$.

Substituting Eqs (18) and (19) into Eq 17 and integrating Eq 17 from *r*_*w*_→*r*, we have

$$\begin{array}{l} u_{2}(r,z,t)=u_{2}(r_{w},z,t)-\frac{1}{2}\cdot \left(\frac{1}{c_{h}}\frac{\partial \bar{u}(z,t)}{\partial t}-\frac{c_{v}}{c_{h}}\frac{\partial^{2}\bar{u}(z,t)}{\partial z^{2}}\right)\cdot r_{w}^{2}\left(n^{2}-s^{2}\right)\text{ln}(\frac{r}{r_{w}})\\ +\frac{s}{a\cdot \beta }\left(\frac{1}{c_{h}}\frac{\partial \bar{u}(z,t)}{\partial t}-\frac{c_{v}}{c_{h}}\frac{\partial^{2}\bar{u}(z,t)}{\partial z^{2}}\right)r_{w}^{2}\cdot \left\{\text{ln}(\frac{r}{r_{w}})[1-e^{\beta }]+\sum_{k=1}^{\infty }\frac{1}{k\cdot k!}{\left(\frac{\beta }{s}\right)}^{k}[{\left(\frac{r}{r_{w}}\right)}^{k}-1]\right\}\\ \begin{array}{ccccccccccccccc} & & & & & & & & & & & & \left(r_{w}\leq r\leq r_{s},0\leq z\leq H;t>0\right) & & \end{array} \end{array}$$ (20)

in which the infinite series $\sum_{k=1}^{\infty }\frac{1}{k\cdot k!}{\left(\frac{\beta }{s}\right)}^{k}[{\left(\frac{r}{r_{w}}\right)}^{k}-1]$ is convergent everywhere; thus, it is adequate to take the first six terms with an error of less than 1%.

Let *r* = *r*_*s*_ in Eq 20; then, we have

$$\begin{array}{l} u_{2}(r_{s},z,t)=-p_{0}-\frac{1}{2}\cdot \left(\frac{1}{c_{h}}\frac{\partial \bar{u}(z,t)}{\partial t}-\frac{c_{v}}{c_{h}}\frac{\partial^{2}\bar{u}(z,t)}{\partial z^{2}}\right)\cdot r_{w}^{2}\left(n^{2}-s^{2}\right)\text{ln}s\\ \begin{array}{cc} & +\frac{s}{a\cdot \beta }\left(\frac{1}{c_{h}}\frac{\partial \bar{u}(z,t)}{\partial t}-\frac{c_{v}}{c_{h}}\frac{\partial^{2}\bar{u}(z,t)}{\partial z^{2}}\right)\cdot r_{w}^{2}\cdot \left\{\text{ln}s\cdot [1-e^{\beta }]+\sum_{k=1}^{\infty }\frac{1}{k\cdot k!}\beta^{k}[1-\frac{1}{s^{k}}]\right\} \end{array} \end{array}$$ (21)

Substituting the continuity conditions of Eq 11-a into Eq 16, we have

$$\begin{array}{l} u_{1}(r,z,t)=-p_{0}-\frac{1}{2}\cdot \left(\frac{1}{c_{h}}\frac{\partial \bar{u}(z,t)}{\partial t}-\frac{c_{v}}{c_{h}}\frac{\partial^{2}\bar{u}(z,t)}{\partial z^{2}}\right)\cdot r_{w}^{2}\left(n^{2}-s^{2}\right)\text{ln}s+\\ \frac{s}{a\cdot \beta }\cdot \left(\frac{1}{c_{h}}\frac{\partial \bar{u}(z,t)}{\partial t}-\frac{c_{v}}{c_{h}}\frac{\partial^{2}\bar{u}(z,t)}{\partial z^{2}}\right)\cdot r_{w}^{2}\left\{\text{ln}s\cdot [1-e^{\beta }]+\sum_{k=1}^{\infty }\frac{1}{k\cdot k!}\beta^{k}[1-\frac{1}{s^{k}}]\right\}\\ +\frac{1}{2}\cdot \left(\frac{1}{c_{h}}\frac{\partial \bar{u}(z,t)}{\partial t}-\frac{c_{v}}{c_{h}}\frac{\partial^{2}\bar{u}(z,t)}{\partial z^{2}}\right)\cdot \left\{\frac{1}{2}\left[r^{2}-r_{s}^{2}\right]-r_{e}^{2}\text{ln}\left(\frac{r}{r_{s}}\right)\right\}\\ (r_{s}\leq r\leq r_{e},0\leq z\leq H;t>0) \end{array}$$ (22)

Substituting Eqs (20) and (22) into the following integral, the equation for $\bar{u}(z,t)$ can be obtained as follows:

$$\bar{u}(z,t)=-p_{0}+E\cdot \frac{r_{w}^{2}}{\left(n^{2}-1\right)}\cdot [\frac{1}{c_{h}}\frac{\partial \bar{u}(z,t)}{\partial t}-\frac{c_{v}}{c_{h}}\frac{\partial^{2}\bar{u}(z,t)}{\partial z^{2}}]$$ (23)

in which

$$\begin{array}{l} E=[-\frac{1}{2}\left(n^{2}-s^{2}\right)+\frac{s}{a\cdot \beta }(1-e^{\beta })][s^{2}\text{ln}s-\frac{1}{2}(s^{2}-1)]+\\ +\frac{s}{a\cdot \beta }\sum_{k=1}^{\infty }\frac{1}{k\cdot k!}{\left(\frac{\beta }{s}\right)}^{k}[\frac{2}{k+2}(s^{k+2}-1)-s^{2}+1]+\\ +[-\frac{1}{2}{\left(n^{2}-s^{2}\right)}^{2}\text{ln}s]+\left(n^{2}-s^{2}\right)\frac{s}{a\cdot \beta }[(1-e^{\beta }).\text{ln}s+\sum_{k=1}^{\infty }\frac{1}{k\cdot k!}\beta^{k}\left(1-\frac{1}{s^{k}}\right)]\\ +\frac{1}{2}[\frac{1}{4}{\left(n^{2}-s^{2}\right)}^{2}+\frac{1}{2}n^{2}\left(n^{2}-s^{2}\right)-n^{4}\text{ln}(\frac{n}{s})] \end{array}$$ (24)

Let

$$D_{1}=\frac{1}{c_{h}}\cdot \frac{r_{w}^{2}}{\left(n^{2}-1\right)}\cdot (-E)$$ (25-a)

$$D_{2}=\frac{c_{v}}{c_{h}}\cdot \frac{r_{w}^{2}}{\left(n^{2}-1\right)}\cdot (-E)$$ (25-b)

Thus, Eq 23 yields

$$\bar{u}(z,t)=-p_{0}-D_{1}\cdot \frac{\partial \bar{u}(z,t)}{\partial t}+D_{2}\cdot \frac{\partial^{2}\bar{u}(z,t)}{\partial z^{2}}$$ (26)

It can also be proven that it is adequate to take the first six terms of the infinite series in Eq 20 with an error of less than 1%.

In equation E, if only terms containing *n* ^4^,*n* ^2^ *s* ^2^,*s* ^4^,*n* ^3^,*n* ^2^ *s*,*ns* ^2^,*s* ^3^ are reserved and marked as *I*_0_+*I*_1_, it can also be proven that the relative error will not exceed 1%.

Let

$$-E\approx I_{0}+I_{1}$$ (27)

in which I _0_ is the ideal part

$$I_{0}=\frac{1}{2}n^{4}\text{ln}n-(\frac{3}{8}n^{4}-\frac{1}{2}n^{2}+\frac{1}{8})$$ (28)

and I_1_ is the smear part,

$$\begin{array}{l} I_{1}\approx \frac{1}{4}n^{2}(s^{2}-1)-\frac{1}{8}(s^{4}-1)\text{+}\frac{1}{4}(s^{2}-1)-\frac{1}{2}n^{2}s^{2}.\text{ln}s\text{+}\\ \frac{s}{a.\beta }.\left\{(e^{\beta }-1).[n^{2}.\text{ln}s-\frac{1}{2}s^{2}]-(n^{2}-s^{2}).\varphi_{1}-s^{2}.\varphi_{2}\right\} \end{array}$$ (29)

in which

$$\varphi_{1}=\sum_{k=1}^{6}\frac{1}{k.k!}\beta^{k}-\sum_{k=1}^{6}\frac{1}{k.k!}{\left(\frac{\beta }{s}\right)}^{k}$$ (30)

$$\varphi_{2}=\sum_{K=1}^{6}\frac{1}{k.k!}.\frac{2}{k+2}.\beta^{k}-\frac{1}{s^{2}}.\sum_{k=1}^{6}\frac{1}{k.k!}.\frac{2}{k+2}.{\left(\frac{\beta }{s}\right)}^{k}-(1-\frac{1}{s^{2}}).\sum_{k=1}^{6}\frac{1}{k.k!}.{\left(\frac{\beta }{s}\right)}^{k}$$ (31)

Thus, Eqs (25-a) and (25-b) yield

$$D_{1}=\frac{1}{c_{h}}\cdot \frac{r_{w}^{2}}{\left(n^{2}-1\right)}\cdot (-E)=\frac{1}{c_{h}}\cdot \frac{r_{w}^{2}}{\left(n^{2}-1\right)}\cdot (I_{0}+I_{1})=\frac{1}{\bar{c_{h}}}\frac{r_{w}^{2}}{\alpha^{2}}$$ (32)

$$D_{2}=\frac{c_{v}}{c_{h}}\cdot \frac{r_{w}^{2}}{\left(n^{2}-1\right)}\cdot (-E)=\frac{c_{v}}{c_{h}}\cdot \frac{r_{w}^{2}}{\left(n^{2}-1\right)}\cdot (I_{0}+I_{1})=\frac{c_{v}}{\bar{c_{h}}}\frac{r_{w}^{2}}{\alpha^{2}}$$ (33)

in which

$$\alpha^{2}=\frac{n^{2}-1}{I_{0}}=\frac{n^{2}-1}{\frac{1}{2}n^{4}\text{ln}n-(\frac{3}{8}n^{4}-\frac{1}{2}n^{2}+\frac{1}{8})},\bar{c_{h}}=c_{h}\cdot \frac{1}{\left(1+s\zeta \right)},\zeta =\frac{I_{1}}{I_{0}}$$

Eq 26 can be rewritten as

$$D_{1}\frac{\partial \bar{u}(z,t)}{\partial t}-D_{2}\frac{\partial^{2}\bar{u}(z,t)}{\partial z^{2}}\text{+}\bar{u}(z,t)=-p_{0}$$ (34)

Combining Eqs 25-b and 27 yields

$$\left\{\begin{array}{l} \frac{1}{D_{1}}=\bar{c_{h}}{\left(\frac{\alpha }{r_{w}}\right)}^{2}=\frac{1}{1+s\zeta }.c_{h}.{\left(\frac{\alpha }{r_{w}}\right)}^{2}\\ \frac{D_{2}}{D_{1}}=c_{v} \end{array}\right\}$$ (35)

$\bar{u}(z,t)$ can be obtained using Eq 34, and the definite solution is as follows:

$$\left\{\begin{array}{l} D_{1}\frac{\partial \bar{u}(z,t)}{\partial t}-D_{2}\frac{\partial^{2}\bar{u}(z,t)}{\partial z^{2}}\text{+}\bar{u}(z,t)=-p_{0}=u_{w}\\ \bar{u}{|}_{z=0}=-p_{0}\\ \frac{\partial \bar{u}}{\partial z}{|}_{z=0}\\ u_{w}=-p_{0}\\ \bar{u}(z,t){|}_{t=0}=0 \end{array}\right\}$$ (36)

Homogenizing the boundary conditions of the solutions above and using the separation of variables method, we have

$$\bar{u}(z,t)=-p_{0}+p_{0}\cdot \sum_{m=0}^{\infty }\left[\frac{2}{M}]\cdot e^{-\lambda_{m}^{2}t}\cdot \text{sin}(\frac{M}{H}z\right)$$ (37)

in which

$$\lambda_{m}^{2}=c_{v}{\left(\frac{M}{H}\right)}^{2}+\bar{c_{h}}{\left(\frac{\alpha }{r_{w}}\right)}^{2}.$$

Considering that

$$\frac{1}{H}\int_{0}^{H}\text{sin}(\frac{M}{H}z).dz=\frac{1}{M},\begin{array}{cc} & \frac{1}{H}\int_{0}^{H}1.dz \end{array}=1$$

the average pore water pressure can be obtained:

$$\bar{u}(t)=-p_{0}[1-\sum_{m=0}^{\infty }\frac{2}{M^{2}}e^{-\lambda_{m}^{2}t}])$$ (38)

The total average degree of consolidation is as follows:

$$U(t)=\frac{\bar{u}(t)}{\bar{u}(\infty )}=1-\sum_{m=0}^{\infty }\frac{2}{M^{2}}e^{-\lambda_{m}^{2}t}$$ (39)

in which

$$M=\frac{\pi }{2}+m\pi ,\begin{array}{cc} & \left(m=0,1,2,\ldots \right) \end{array}$$

## Results

### Comparison with the previous solution without the smear effect

The solution by Rujikiatkamjorn et al.[11] and a numerical solution are presented to verify this study's solution. Some of the parameters used in the analysis are provided in Table 1.

**Table 1:** Parameters used in the analytical solution and the numerical model.

Parameters	Value
*r*_*e*_ (m)	0.75
*H* (m)	5
*s = d*_*s*_ */d*_*w*_	4
*r*_*w*_ (m)	0.075
*n = d*_*e*_ */d*_*w*_	10
C_v_ (cm^2^/s)	4.76e-4
C_h_ (cm^2^/s)	9.51e-4
*k*_*h*_ of the soil (m/s)	3.80e-10
*k*_*v*_ of the soil (m/s)	1.90e-10
Oedometric modulus, *E*_*0*_ (MPa)	1.857
Poisson’s ratio	0.3

**Fig 3:**
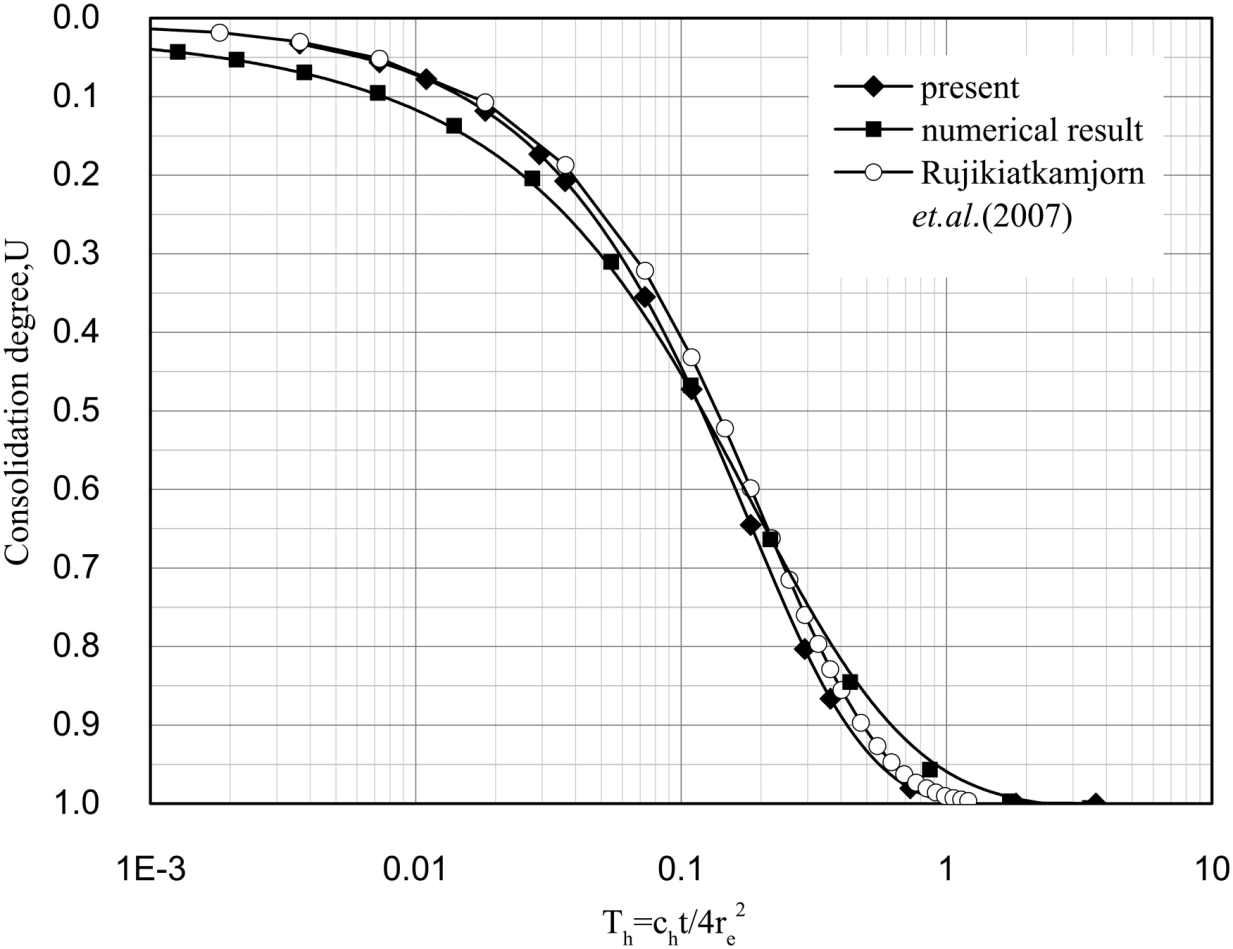
Solutions of Rujikiatkamjorn et al.[11], the present model, and the numerical analysis.

In the solution by Rujikiatkamjorn et al.[11], well resistance was not considered, and the permeability coefficient in the smear zone was a reduced constant. When the smear effect is not considered (i.e., let s = 1, *ζ* = 0), this study's solution is nearly the same as the solution of Rujikiatkamjorn et al.[11] (Fig 3).

A numerical model based on Eq 5 is developed to analyze the consolidation of soil treated by vacuum preloading with a central PVD. The equal strain hypothesis is not necessary in the numerical model. The parameters adopted in the numerical model are displayed in Table 1.

The comparison is shown in Fig 3. The results from the analytical solution derived in this study correlate well with those from the numerical simulation, although there are a few differences between the two solutions, the difference between the analytical solution and numerical solution is attributed to the hypothesis of equal strain of analytical solution, which undervalued the degree of consolidation in the early period, and corresponding overvalued the consolidation degree in the later period. The difference also can be found in literatures[27,28].

### Comparison with the previous solution with the nonlinear smear effect

**Fig 4:**
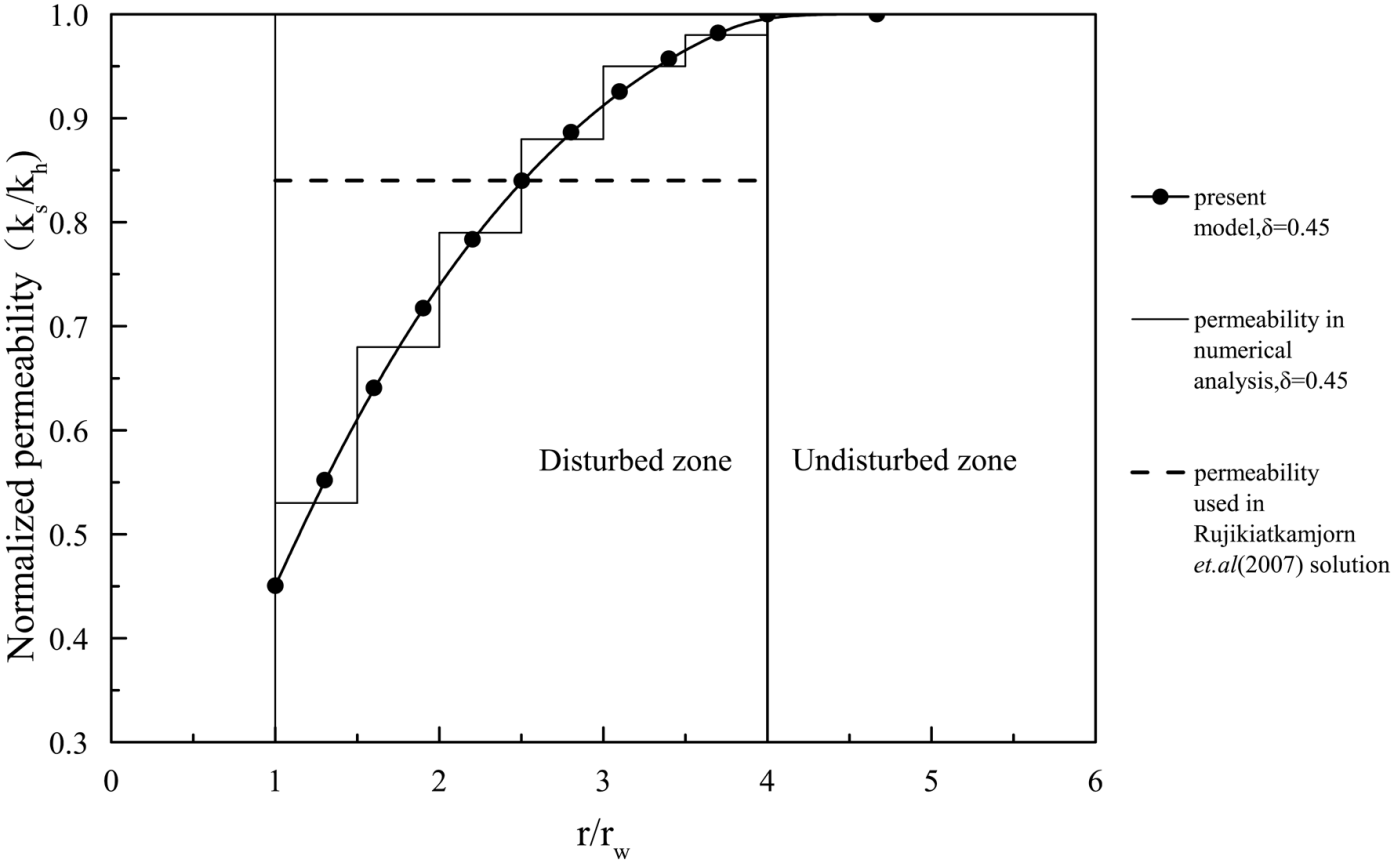
Permeability coefficients of the present model and numerical analysis (δ = 0.45) and of the study by Rujikiatkamjorn et al.[11].

**Fig 5:**
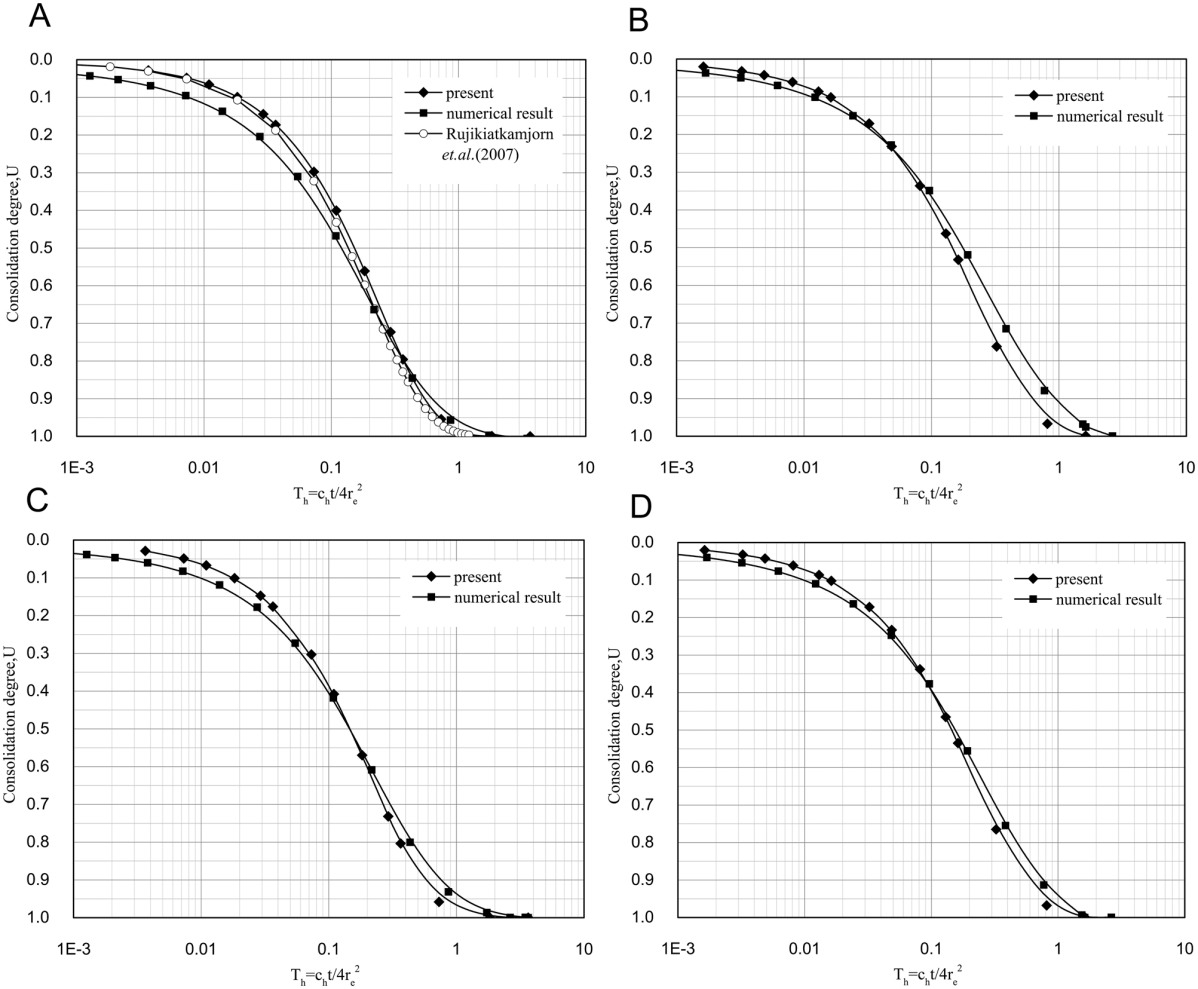
Comparison of this study's solution and the numerical solution for different values of *δ and n*. (A) δ = 0.45, n = 10. (B) δ = 0.45, n = 15. (C) δ = 0.55, n = 10. (D) δ = 0.55, n = 15.

The solution with the nonlinear smear effect is also validated. The nonlinear change in the permeability coefficient within the smear zone is shown in Fig 4. Some of the parameters used in the analysis are provided in Table 1, and the consolidation degree is provided in Fig 5(a).

There are no other similar analytical solutions that consider the nonlinear change of the permeability coefficient within the smear zone, so the numerical solution is presented for comparison first. The permeability coefficient within the smear zone is simulated by segmented constants in the numerical solution, as shown in Fig 4. The consolidation degree of the numerical solution is shown in Fig 5(a), as well as the comparison with the proposed analytical solution. The results from the analytical solution derived in this study correlate well with the numerical simulation.

Rujikiatkamjorn et al.[11] assumed that the permeability coefficient of the smear zone was a constant less than that of the undisturbed zone, as shown in Fig 4. The weighted average permeability coefficient of this sample is *k*_*hs*_ = 0.84*k*_*h*_, which yields the consolidation degree shown in Fig 5(a) when substituted into the solution by Rujikiatkamjorn et al.[11]. The solution of Rujikiatkamjorn et al.[11] is close to this study's solution, but its consolidation speed is faster than both this study's solution and the numerical solution in the latter period. This discrepancy means that the consolidation speed is affected by the non-uniform change in permeability within the smear zone. The permeability of the smear zone is overestimated when an average permeability coefficient is adopted. The results show the presented solution is more precise than the solution with constant permeability in smear zone.

**Fig 6:**
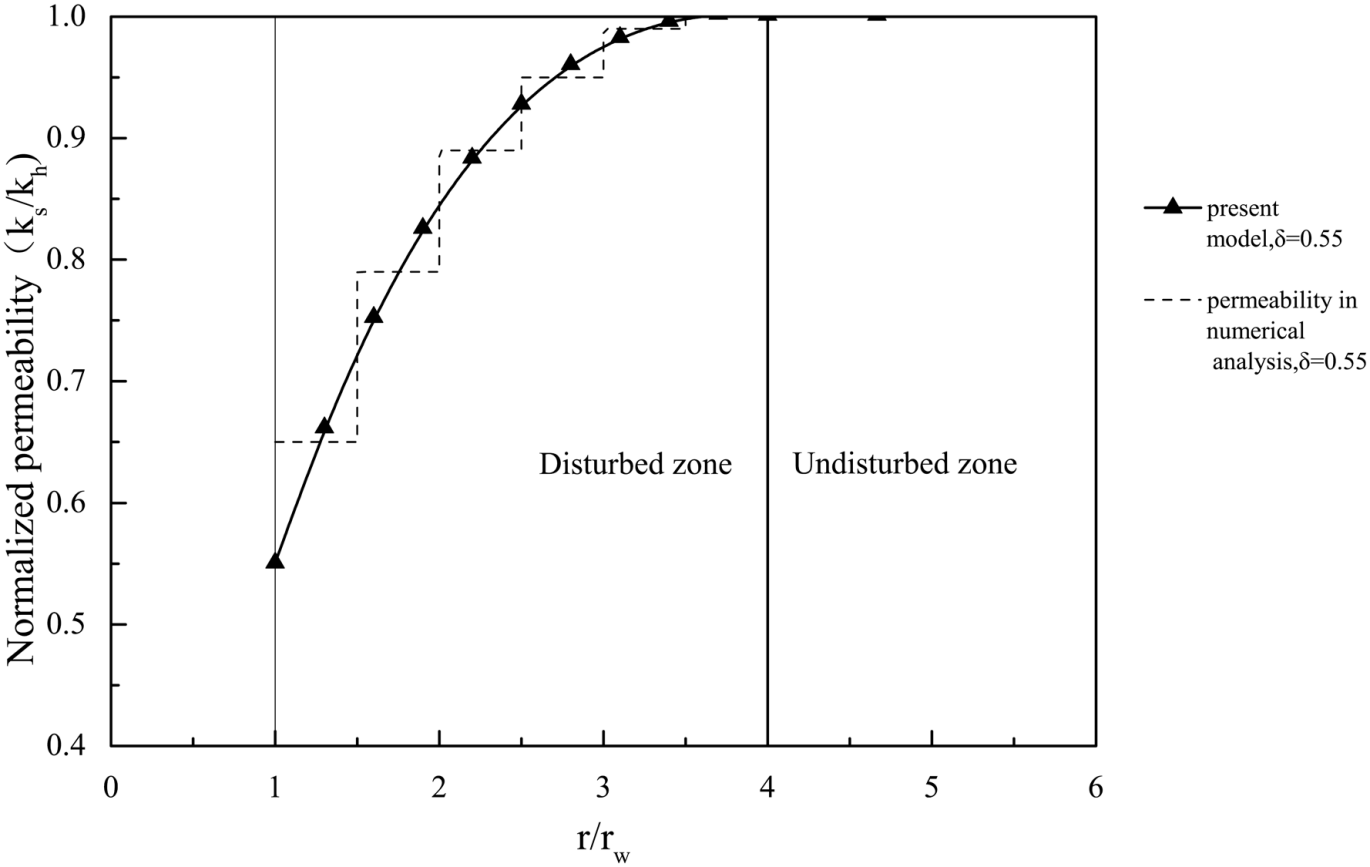
Permeability coefficient of the present model and the numerical analysis when δ = 0.55.

The comparisons of this study's solution and the numerical solution when s = 4, δ = 0.55, and n = 10 or 15 are shown in Fig 5b–5d. The permeability coefficient within the smear zone when δ = 0.55 is simulated by segmented constants in the numerical solution, as shown in Fig 6. There is little difference between the two solutions, and the trend is consistent, which indicates that this study's solution correlates well with the numerical solution.

## Conclusion

In this study, a vertical drain radial consolidation equation of vacuum preloading is presented, the nonlinear reduction of permeability in smear zone is modeled by exponential function, and the consolidation equation is solved by separation of variables method. The validity of the solution has been evaluated by comparison with previous analytical solution and numerical simulation, the result shows the analytical solution presented in this study correlates well with the numerical simulation and is more precise than previous analytical solution with considering constant permeability in smear zone.

## Nomenclature

The following symbols are used in this article:

*E*, *I*_*0*_, *I*_*1*_, *D*_*1*_, *D*_*2*_, *φ*_*1*_, *φ*_*2*_,*λ*_*m*_,*ζ*,*α*, *M* parameters of *u*

*a*, *β* Coefficients of the permeability function in the smear zone

*γ*_*w*_ Specific weight of water

*c*_*h*_, *c*_v,_ Horizontal and vertical coefficients of consolidation

*E*_*0*_ Oedometric modulus

*δ* Ratio of the permeability coefficient at the drain-soil interface to that of the undisturbed zone

*H* Depth of soil

*k*_*h*_, *k*_*v*_ Horizontal and vertical permeability coefficients of the soil

*m*_*v*_ Coefficient of volume compressibility of the soil

*n* Ratio of the radius of the model to the drain

-*p*_0_ Value of the vacuum pressure

*s* Ratio of the radius of the smear zone to that of the drain

*r* Radial position

*r*_*s*_ Radius of the smear zone

*r*_*w*_ Radius of the drain

*r*, *z* Radial and vertical coordinates

*t* Time

*u* Excess pore water pressure in the soil

*u*_*1*_ Excess pore water pressure in the smear zone

*u*_*2*_ Excess pore water pressure in the undisturbed zone

*u*_*w*_ Excess pore water pressure in the drain

$\bar{u}$ Average excess pore water pressure in the soil

*U* Average degree of consolidation in the entire model

## Supporting Information

S1 Fig Data of Fig 3. (XLS)

S2 Fig Data of Fig 5(A). (XLS)

S3 Fig Data of Fig 5(B). (XLS)

S4 Fig Data of Fig 5(C). (XLS)

S5 Fig Data of Fig 5(D). (XLS)
